# Integrative Analysis of Prognostic Value and Immune Infiltration of Spindle and Kinetochore-associated Family Members in Breast Cancer

**DOI:** 10.1080/21655979.2021.1995576

**Published:** 2021-11-30

**Authors:** Jianfeng Ding, Xiaobo He, Jinkun Wang, Guodong Cao, Sihan Chen, Liping Yuan, Bo Chen, Maoming Xiong

**Affiliations:** a Department of General Surgery, The First Affiliated Hospital of Anhui Medical University, Hefei, Anhui, China; b Department of General Surgery, Chaohu Hospital of Anhui Medical University, Chaohu, Anhui, China; c Department of Pediatrics, The First Affiliated Hospital of Anhui Medical University, Hefei, Anhui, China

**Keywords:** Spindle and kinetochore associated complex subunit, breast cancer, prognosis, immune infiltrates, biomarker

## Abstract

Spindle and kinetochore associated (SKA) complex subunit, which maintains the stability of mitotic metaphase, with emerging research implying its effect as a carcinogenic regulator in cancer. However, its potential role in BC has not been fully elucidated. ONCOMINE, UALCAN, GEPIA, Kaplan-Meier Plotter, cBioPortal and TIMER databases were performed to analyze the expression, prognosis, mutation, immune infiltration and potential biological mechanisms of SKA1/2/3 in BC. Our results showed that SKA1/2/3 expression was upregulated in BC. Survival analysis reveals that SKA3 overexpression was associated with poor overall survival (OS), relapse-free survival (RFS), post-progression survival (PPS) and distant metastasis-free survival (DMFS). SKA1 overexpression was associated with poor OS, RFS and DMFS while SKA2 overexpression was only associated with RFS and DMFS. Notably, the results implied that SKA1 has a good prognostic value in HER2-positive BC. Besides, the genetic alterations of SKA were investigated and the altered group correlated with shorter progress-free survival (PFS) and disease-specific survival (DSS). GO and KEGG analysis showed that SKA1/2/3 were implicated in regulating cell cycle, p53 signaling pathway and DNA replication. The 10 Hub genes in the protein network were upregulated in BC and related to poorer prognosis. Additionally, SKA1/2/3 expression was negatively correlated with infiltration of various immune cells with antitumor effects, whereas positively correlated with the expression of immune checkpoints molecules. Further experiments revealed that SKA1/2/3 silencing markedly impeded the proliferation and migration of BC cells. Herein, our study firmly shows that SKA genes may serve as a promising therapeutic target for patients with BC.

## Introduction

Breast cancer (BC) remains the most aggressive malignancy for women worldwide and is related to a high mortality rate. Global cancer statistics report that BC accounts for approximately 30% of all new female cancer cases diagnosed in 2019 [1]. Despite the development of various treatments for BC during the last decades, more than 20% of BC patients still face the dilemma of tumor recurrence [2]. Therefore, exploring more potential molecular targets underneath BC progression is of great significance to develop novel prognosis or therapeutic biomarkers for BC.

The spindle and kinetochore associated (SKA) subunit complex, composed of two copies of SKA1, SKA2, and SKA3 proteins, a microtubule-binding protein of the outer kinetochore that is indispensable to stabilize spindle microtubules attaching to the kinetochore (KT) and maintain accurate chromosome segregation in the metaphase of mitosis [3–5]. Notably, SKA1/2/3, as new cancer-related genes, has been implicated in the progression of numerous cancer types. High level of SKA1 contributed to the proliferation, migration, and metastasis of pancreatic ductal adenocarcinoma cells and reflected poor overall survival [6]. SKA2 was highly expressed in hepatocellular carcinoma (HCC) and can be used as a potential tumor promoter for HCC [7]. Moreover, SKA3 overexpression was associated with chromosomal instability, while interference with SKA3 in colorectal adenoma cells induced G2/M arrest, reduced migration and invasion abilities, and increased apoptosis [8]. Nevertheless, the role of SKA members in BC remains elusive. Therefore, it is urgent to explore the potential carcinogenic capacity of different SKA family members in tumors.

With the development of high-throughput sequencing technology and the emergence of various friendly database websites, bioinformatics analysis is increasingly applied to explore the role of genes in cancer. The present study was designed to determine the expression, prognosis, clinicopathological features, mutation, potential regulatory mechanisms, and immune infiltration of SKA1/2/3 in BC patients. Further in vitro experiments demonstrate the functional role of the SKA genes in the progression of BC. In general, this study may contribute to providing a foundation for more understanding the molecular basis of SKA-mediated BC development, thereby providing valuable information on whether SKA1/2/3 could be used as potential tumor-promoting regulators of BC.

## Materials and methods

### Oncomine analysis

Oncomine (www.oncomine.org), an online datamining platform and cancer microarray database, was conducted to analyze transcriptional levels of SKA family members in distinct tumors and normal control tissues [9]. The student’s t-test was applied to get the p-value, taking p-value<0.001 and fold change = 2 as a threshold.

### UALCAN and GEPIA analysis

UALCAN (https://ualcan.path.uab.edu/index.html) and GEPIA (http://gepia.cancer-pku.cn/index.html) were user-friendly online websites for TCGA data mining analysis, which can be used for various in-depth analysis of multiple genes [10,11]. In this study, UALCAN and GEPIA were performed to determine the differential expression levels of SKA gene members and their co-expressed Hub genes in normal and tumor tissues, pathological stages, and various molecular subtypes of BC. The p-value for all of the above differential expression analysis was calculated following the student’s t-test. P < 0.05 was regarded as statistically significant.

### Kaplan-meier plotter

The Kaplan-Meier Plotter (https://kmplot.com/analysis/), a publicly accessible website including gene expression data and survival information of patients with breast, gastric, liver, lung cancer, etc., was performed to predict the prognosis of SKA members and 10 Hub genes in BC [12]. Based on the median value of mRNA expression, patients were split into low and high expression cohorts. Overall survival (OS), relapse-free survival (RFS), post-progression survival (PPS), and distant metastasis-free survival (DMFS) of patients with BC were assessed by Kaplan-Meier survival plot and Log-rank test. It was considered to have statistical significance when p-value < 0.05.

### cBioPortal analysis

cBioPortal (www.cbioportal.org), an interactive web application to explore and visualize multidimensional cancer genomics data, was established to analyze the genetic alterations and copy number variations (CNVs) of SKA members in BC, and the correlation between mutation and survival (OS, PFS, and DSS) [13]. The Breast Invasive Carcinoma (TCGA, PanCancer Atlas) dataset which contained 1084 patients were selected for alteration analysis and the p-value was calculated by the Log-rank test.

### The cancer cell line encyclopedia

The Cancer Cell Line Encyclopedia (CCLE) project is an effort to conduct a detailed genetic characterization of a large panel of human cancer cell lines. The cell line expression matrix of pan-cancer was obtained from the CCLE dataset (https://portals.broadinstitute.org/ccle/about) [14].

### Bioinformatic analysis

RNA-sequencing expression data and corresponding clinical information of 1178 BC patients were downloaded from The Cancer Genome Atlas (TCGA) dataset (http://cancergenome.nih.gov/). Univariate and multivariate Cox analysis were used to determine SKA1/2/3 as independent prognostic factors for OS in BC. Additionally, the KM survival analysis with the log-rank test was also used to compare the survival difference between high- and low-expression levels of SKA1/2/3 in HER2-positive BC. TimeROC analysis was performed to compare the predictive accuracy of each gene. The co-expression genes of SKA members were obtained through Spearman correlation coefficient analysis (cor>0.4, p-value<0.001). Gene ontology (GO) and Kyoto Encyclopedia of Genes and Genome (KEGG) analysis were conducted on co-expression genes employing the ‘clusterProfiler’ package to evaluate potential biological functions and signaling pathways. String Database (https://string-db.org/) was conducted to establish a protein-protein interaction (PPI) network for SKA co-expression genes, and the cutoff of the minimum required interaction score was 0.7 [15]. The number of interconnections of SKA co-expression genes in the PPI network based on the R package was calculated to screen Hub genes for further analysis.

### TIMER2.0 database analysis

Timer (http://timer.cistrome.org/), an online tool for comprehensive analysis of immune infiltrates across multiple tumors, was used to evaluate the correlation between SKA1/2/3 and distinct immune cell infiltration in BC [16]. The correlation scatterplots between the mRNA expression of SKA1/2/3 and several common immune checkpoints were obtained via the ‘Correlation module’ on the website.

### Cell lines and cell culture

Human BC cell lines (MCF-7, T47D, MDA-MB-231) and the normal human cell line (MCF10A) were provided from the Type Culture Collection of the Chinese Academy of Sciences (Shanghai, China). Human breast epithelial cells (MCF-10A) were cultured in DMEM/F12 supplemented with 10% fetal bovine serum (Gibco; Thermo Fisher Scientific, Inc.) and maintained at 37°C in a 5% CO2 atmosphere for cultivation. Human BC cell lines (MCF-7, T47D, MDA-MB-231) were cultivated in DMEM with 10% FBS under the same conditions.

### Quantitative real-time polymerase chain reaction (qRT-PCR)

Reverse transcription of the RNA isolated from cultured cells and real-time quantitative (qRT-PCR) were performed following the manufacturer’s protocol of RT-PCR kit (Life technogies) and the fluorescence quantitative PCR kit (TaKaRa). Primer sequences for qRT-PCR were as follows: SKA1 forward: CTTGAGGTTGGAGTCTGTGT, SKA1 reverse: TTGTTCCAGATCTGACGAGG; SKA2 forward: AAAGTGAAGGAGATCTACTGGG, SKA2 reverse: TGTAATCCAGATCAGACTCAGC; SKA3 forward: AGGACCTAGCATACTGTGGA, SKA3 reverse: TCCCAAAACTACGCCTTTGA. Relative gene expression was determined via the 2^−ΔΔCt^ method.

### Western blot analysis

Total proteins were extracted and quantified from cultured cells utilizing lysis buffer and the Pierce BCA protein assay kit (Thermo, USA), respectively. The protein extracts (20 mg) were separated by SDS-PAGE and transferred to PVDF membranes. The membranes were blocked 5% nonfat milk buffer, and then probed with primary antibodies against SKA1 (dilution: 1:1000, ab91550), SKA2(dilution: 1:1000, ab75345), and SKA3(dilution: 1:1000, ab186003) which were all purchased from Bioss Company. After incubation with secondary anti-rabbit antibodies or anti-mouse, protein bands were visualized using an enhanced chemiluminescence reagent kit (Thermo, USA). The relative expressions of SKA1/2/3 were detected employing a chemiluminescence detection system in line with the manufacturer’s instructions.

### Cell viability assay

BC cell proliferation was quantified by Cell Counting Kit-8 (CCK-8; Dojindo, Tokyo, Japan) assay in this study. Cells were added to 96-well plates at a density of 3x10^3^/well and incubated for 1 to 5 days. Cell viability was measured using CCK-8 according to the manufacturer’s protocol at an optical density of 450 nm using an automated plate reader (Bio-Rad, USA).

### Wound-healing assay

The cells were seeded in 6-well plates and cultured to 100% confluence at 37°C with 5% CO2. A 100 µL plastic tip was used to create an artificial wound. After washing with phosphate buffer saline, cells were incubated in fresh medium with 1% FBS. Images were taken at 0 and 24 h to assess the distance of migration. Cell mobility = (0 h width − 24 h width)/0 h width × 100%.

### Statistical analysis

The statistical analyses were conducted using GraphPad Prism 8 (GraphPad Software Inc., USA). The results are shown as mean ± standard deviation (SD) and were compared via Student’s t-test or one-way ANOVA. Kaplan–Meier plot survival curve was analyzed using the log‐rank test. The correlation coefficients were calculated by the Spearman test. P < 0.05 was regarded as statistically significant.

## Results

The present study explored the expression and prognostic value of SKA family genes. In addition, SKA family genes were further subjected to mutation and immune infiltration correlation analysis, as well as co-expressed genes of SKA family genes were extracted to investigate their underlying regulatory mechanisms in tumorigenesis and tumor development. Moreover, in vitro experiments highlight the role of SKA family genes in promoting tumor proliferation and migration. Overall, this study indicated that SKA1/2/3 act as potential oncogenes in BC.

### Differential expression of SKA1/2/3 in pan-cancer

**Figure 1.**
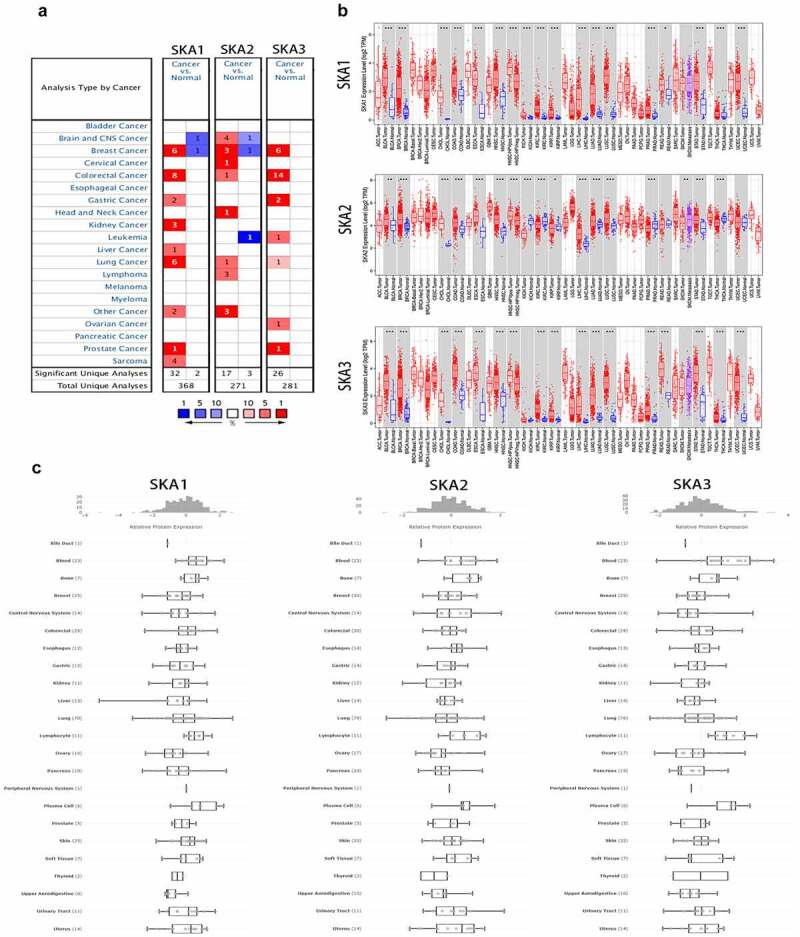
**The mRNA and protein expression levels of SKA1/2/3 among distinct types of cancers**. (a) Analyses were conducted in Oncomine. Cells in red color represents the overexpression of interested gene, while downexpression shows in blue. The numbers in these cells represents the numbers of datasets which satisfies the threshold: p-value: 0.001, fold change: 2. The gene rank was analyzed by percentile of target gene in the top of all genes measured in each research. Cell color is determined by the best gene rank percentile for the analyses within the cell. (b) Analyses were conducted in TIME2.0. Red represents the expression level of SKA1/2/3 in tumors and blue represents the expression level of SKA1/2/3 in para-tumor tissues. (c) Protein expression profile of SKA1/2/3 in cell lines of various cancers in CCLE database. *P < 0.05, **P < 0.01, and ***P < 0.001

The expression levels of SKA1/2/3 were detected between tumors and normal tissues in Oncomine and Timer databases. According to the Oncomine database, we found that the transcriptional levels of SKA1/2/3 were highly expressed in various tumor types, including BC (Figure 1(a) and Table 1). Meanwhile, the results in the Timer database also verified that the expressions of SKA1/2/3 were generally upregulated in Pan-cancer (Figure 1(b)). Furthermore, we explored the protein expression profile of SKA1/2/3 in cell lines of various cancers in the CCLE database. A total of SKA member genes was found that are expressed at higher levels in the BC cell lines (Figure 1(c)).

**Table 1.** Significant changes of SKA1/2/3 expression in transcription level between BC and normal tissues (ONCOMINE database)

	Types of breast cancer vs. normal	Fold Change	t-Test	P-value	Source and/or Reference
SKA1	Invasive ductal breast carcinoma	7.501	6.131	3.48E-6	Turashvili Breast
	Invasive ductal breast carcinoma	4.441	21.163	4.31E-40	TCGA
	Invasive breast carcinoma	3.770	12.292	1.18E-23	TCGA
	Male breast carcinoma	2.118	7.305	5.77E-6	TCGA
	Invasive lobular breast carcinoma	2.456	8.295	3.72E-12	TCGA
	Ductal breast carcinoma	2.431	6.641	2.43E-7	Richardson Breast
SKA2	Ductal breast carcinoma in situ epithelia	3.044	9.404	1.10E-7	Ma Breast
	Invasive ductal breast carcinoma epithelia	3.582	5.796	8.50E-6	Ma Breast
	Ductal breast carcinoma	2.066	6.080	1.94E-7	Richardson Breast
SKA3	Intraductal cribriform breast adenocarcinoma	7.598	26.475	8.59E-36	TCGA
	Male breast carcinoma	6.216	22.359	8.42E-26	TCGA
	Invasive breast carcinoma	6.760	16.575	1.23E-34	TCGA
	Invasive ductal breast carcinoma	3.529	23.552	2.23E-45	TCGA
	Invasive lobular breast carcinoma	4.627	10.979	1.28E-16	TCGA
	Mixed Lobular and Ductal Breast Carcinoma	4.924	5.827	3.19E-4	TCGA

### SKA1/2/3 were highly expressed in BC and significantly correlated with clinicopathological features of patients

**Figure 2.**
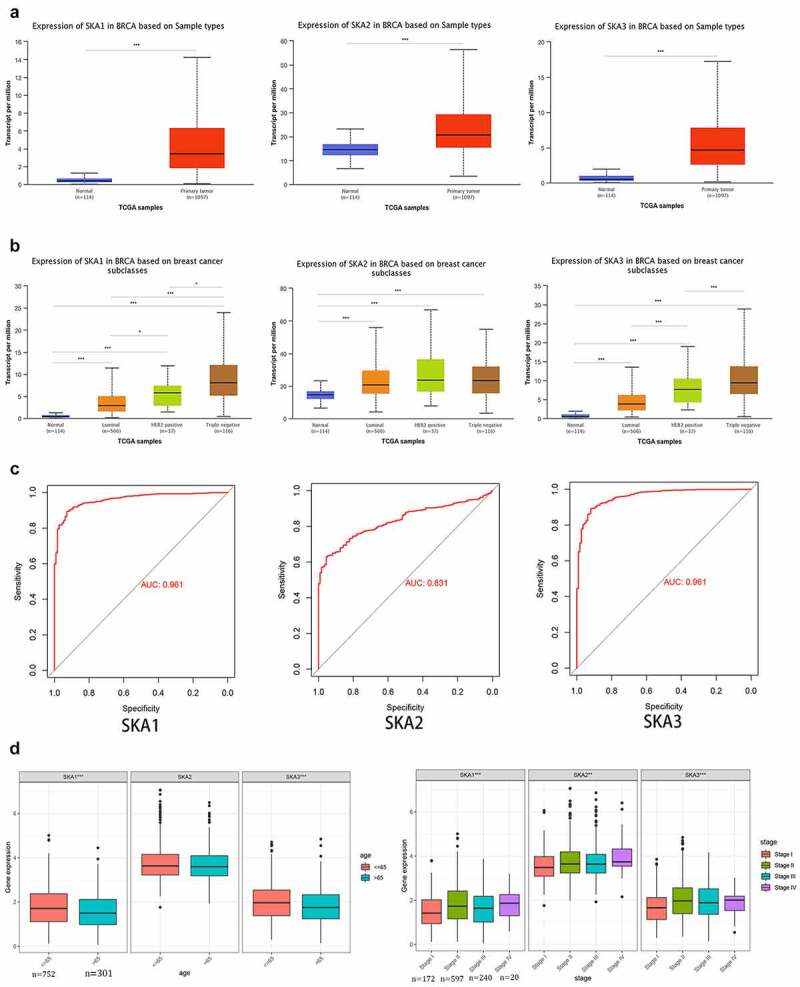
**SKA1/2/3 was overexpressed in BC and correlated with clinicopathological features of patients** (a) The expression levels of SKA1/2/3 between normal and tumor tissues in Ualcan database. (b) SKA1/2/3 expression in different molecular subtypes of BC in Ualcan database. (c) The diagnostic value of SKA1/2/3 in patients with BC. (d) The expression levels of SKA1/2/3 were correlated with the age and tumor stage of BC patients. *P < 0.05, **P < 0.01, and ***P < 0.001

In addition, UALCAN dataset was carried out to analyze the expression levels of SKA1/2/3 in BC. As shown in Figure 2(a), the mRNA expression of SKA1/2/3 in tumor tissues was significantly higher than that in normal tissues, and the AUC was 0.961, 0.831, and 0.961, respectively (Figure 2(c)). Of interest, it was found that the expression levels of SKA1/3 among different molecular subtypes of BC are the highest in Triple-negative, followed by HER2 positive and luminal (Figure 2(b)).

Considering that SKA1/2/3 may be implicated in the development and progression of BC, we further assessed the relationship between the SKA1/2/3 expression and the clinicopathological features (age and tumor stage) for patients with BC. The results showed that the expression levels of SKA1/2/3 were increased accompanying a higher pathological stage (P < 0.05) and it is also noteworthy that the SKA1/3 expression was upregulated in patients younger than 65 years compared to those older than 65 years (P < 0.05) (Figure 2(d)).

### Prognostic value of SKA1/2/3 in BC

**Figure 3.**
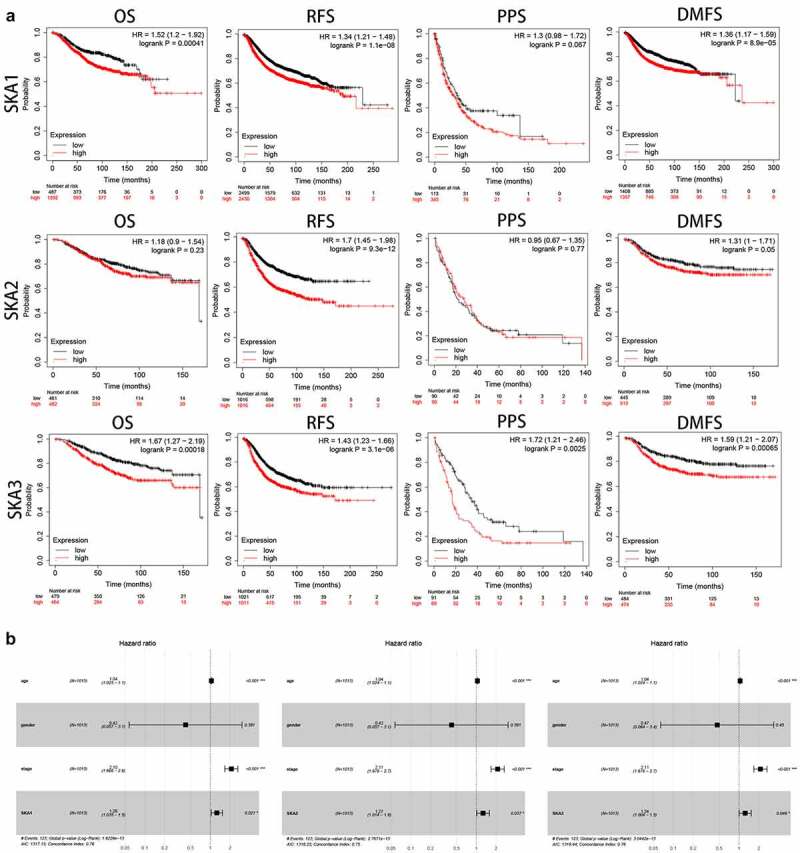
**The prognostic value of SKA1/2/3 expression in BC patients**. (a) High level of SKA1 was associated with shorter OS, RFS and DMFS while high level of SKA2 was only associated with shorter RFS and DMFS. High level of SKA3 was associated with shorter OS, RFS, PPS and DMFS. (b) Multivariate Cox analyses evaluating the independent prognostic value of the SKA1/2/3 in terms of OS in BC patients

To explore the prognostic value of SKA1/2/3 in BC patients, kaplan-Meier plotter database was performed to evaluate the correlation between survival time and different expression levels of SKA1/2/3. Figure 3(a) showed that high levels of SKA1/3 were significantly related to poor OS, RFS, and DMFS in BC patients. Furthermore, the SKA3 overexpression indicated worse PPS and conversely, SKA1 displayed no statistical differences. Additionally, the high level of SKA2 was only obviously associated with the poorer RFS and DMFS, whereas no statistical difference in OS and PPS. Cox analyses were then applied to evaluate the independent prognostic value of SKA1/2/3 in terms of OS of BC patients. Multivariate Cox survival analysis revealed that SKA1/2/3 were independently associated with significantly poorer OS of BC patients (HR>1, P < 0.05), which could serve as independent prognostic factor for BC (Figure 3(b)). Taken together, combined with the survival analysis of SKA1/2/3, our results suggested that SKA1/3 have a strong prognostic value for BC patients.

### SKA1 was associated with clinicopathological features and overall survival in HER2-positive BC

**Figure 4.**
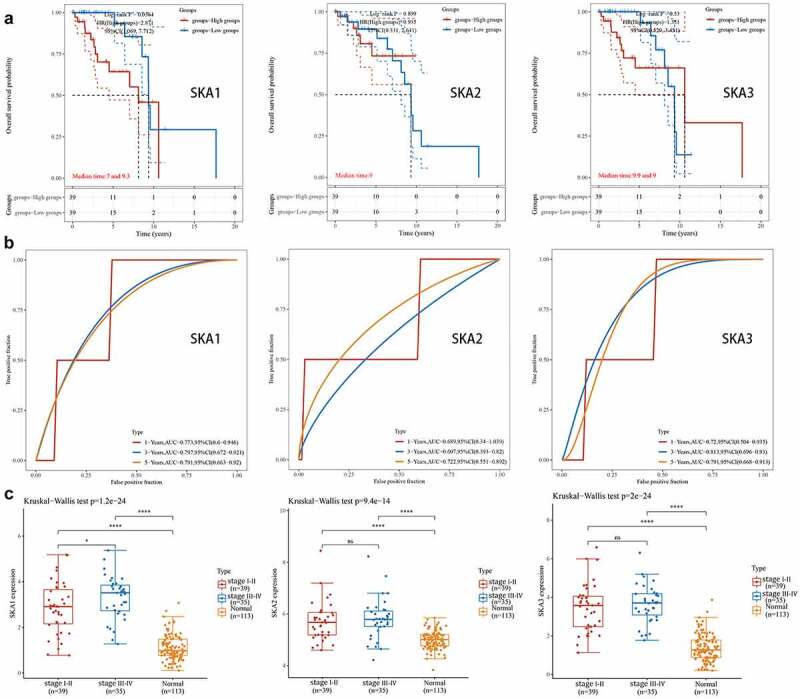
**The analysis of clinicopathological features and prognostic value of SKA1/2/3 in HER2-positive BC**. (a) Kaplan-Meier survival analysis of SKA1/2/3 in TCGA database. Median time represents the time (i.e. median survival time) corresponding to the survival rate of 50% in the high expression group and low expression group. (b) ROC curve showing the prognostic value of SKA1/2/3 on the 1-, 3-, and 5-years survival rate. (c) The expression distribution of SKA1/2/3 in tumor tissues and normal tissues, where the horizontal axis represents different groups of samples, the vertical axis represents the gene expression distribution

Among patients with metastatic breast cancer, more than 20% have HER2-positive disease. In a previous study, we found that SKA1/2/3 was significantly highly expressed in HER2-positive BC (Figure 2(b)), so we further investigated the clinical relevance and prognostic value of SKA1/2/3 among HER2-positive BC patients. The results showed that SKA1 was expressed at a higher level in the late compared with the early stage group (Figure 4(c)). In addition, overall survival indicated that patients with high SKA1 expression have a significantly worse prognosis (P = 0.0364, HR = 2.871, 95%CI: 1.069–7.712), and the AUCs of 1-, 3-, and 5-year clinical outcomes were separately 0.773, 0.797, and 0.791 (Figure 4(a,b)). However, the analysis regarding SKA2/3 in HER2-positive BC showed no statistical significance. Overall, the above results indicate a good predictive efficacy of SKA1 in HER2-positive BC patients.

### Alteration analysis of SKA1/2/3

**Figure 5.**
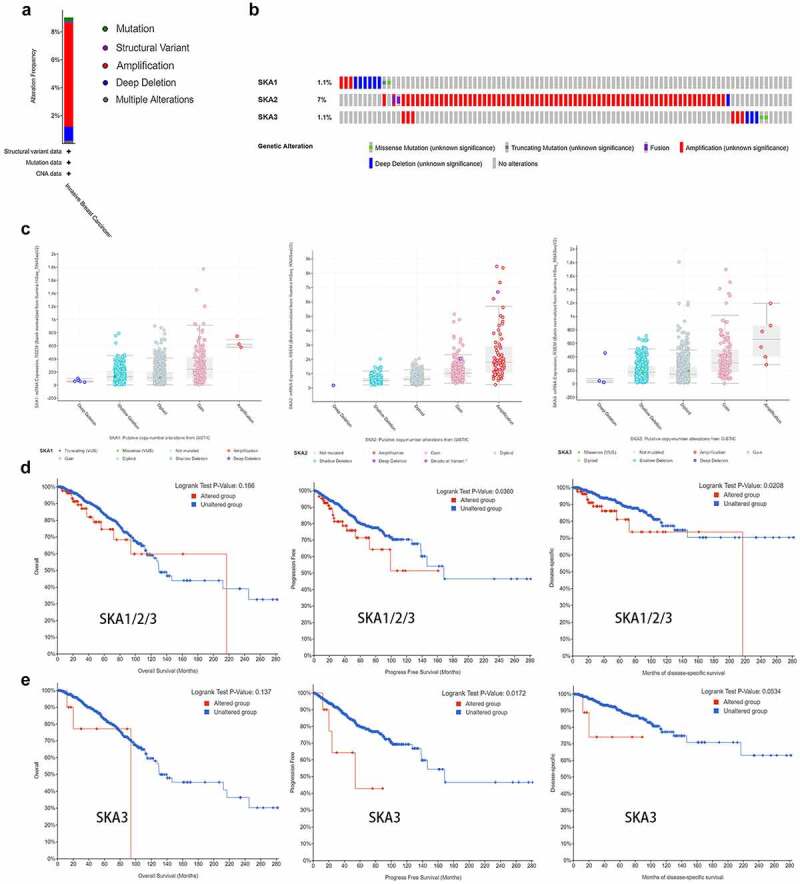
**Genomic alterations and prognostic analysis of SKA1/2/3 in BC**. (a-b) Summary of genetic alterations of SKA1/2/3 in BC. 90 samples of 996 patients were involved in alternations of SKA1/2/3, which account for 9.04%. (c) SKA1/2/3 expression in different CNV groups (d-e) K–M plots curve of OS, PFS and DSS in BC patients with/without the SKAs/SKA3 alterations. Analyses were conducted in cBioPortal

We utilized cBioPortal tool to further investigate the genetic alterations of SKA1/2/3 and their correlations with overall survival (OS), progress-free survival (PFS), and disease-specific survival (DSS) of BC patients. As for the Breast Invasive Ductal Carcinoma (with 996 cases), which 90 cases (9.04%) involved in genetic alternations of SKA1/2/3 [3 cases (0.3%) of mutation, 1 case (0.1%) of structural variant, 74 cases (7.43%) of amplification, 10 cases (1%) of deep deletion, 2 cases (0.2%) of multiple alterations] (Figure 5(a)). Besides, the alteration frequency of SKA1/2/3 was 1.1%, 7%, and 1.1% of the queried BC patients, respectively (Figure 5(b)). Amplifications were the most common alteration characteristic and deep deletions occurred in all SKA members. Furthermore, box plot analysis of CNV data from the BC patients revealed that increased copy number of the genes, especially for the gene amplification, correlated with higher expression of SKA family (Figure 5(c)).

Additionally, Figure 5(d) showed that the patients with genetic alterations in the SKA family had worse PFS and DSS, but not with OS. For more clear interpretation, Kaplan–Meier analyses were re-performed for each gene. We found that patients with SKA3 alteration were associated with poor PFS and DSS (Figure 5(e)), while SKA1/2 showed no significant difference between the altered group and the unaltered group (data not shown). The results demonstrated that genetic alterations of SKA3 may affect the prognosis of BC patients.

### Identification of co-expressed genes with SKA1/2/3 and their Hub genes

**Figure 6.**
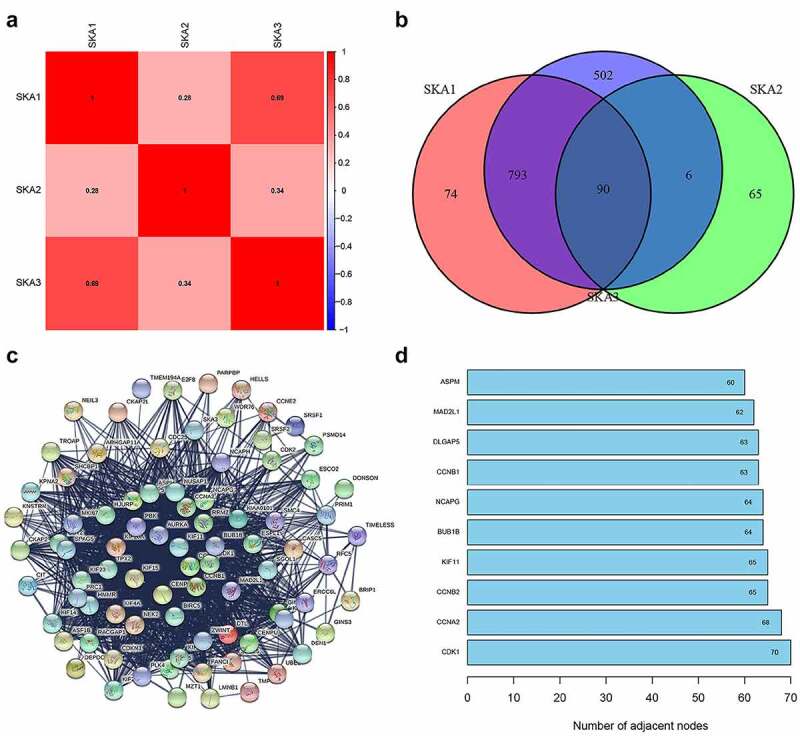
**Genes co-expressed with SKA1/2/3**. (a) Spearman correlation analysis of SKA1/2/3 in TCGA databases. (b) Venn diagram visualizing the intersection co-expressed genes of SKA1/2/3, |cor| >0.4 and P < 0.001. (c) Protein–Protein Interaction interactions among 90 overlapping genes correlated with co-expressed genes of SKA1/2/3. (d) The 10 genes with the highest interaction degrees were labeled in the protein network

We obtained RNA-sequencing data of SKA1/2/3 from the TCGA database to further understand their regulatory relationship, and the results showed that they were obviously positively associated with one another, especially between SKA1 and SKA3 (r = 0.69) (Figure 6(a)).

To better understand the role of SKA members in BC, we obtained the co-expressed genes of SKA1/2/3 in accordance with the screening criteria. The results indicated that there were 1275 positively and 325 negatively correlated genes with SKA1, 161 positively and 0 negatively correlated genes with SKA2, and 1105 positively and 284 negatively correlated genes with SKA3. As seen in the Venn diagram, 90 overlapping genes correlated with co-expressed genes of SKA1/2/3 were identified in BC (Figure 6(b)).

In addition, a string online tool was used to conduct a protein-protein interaction network for these 90 overlapping genes to observe their interactions (Figure 6(c)). The 10 genes with the highest interconnection levels were maintained, including CDK1, CCNA2, CCNB2, KIF11, BUB1B, NCAPG, CCNB1, DLGAP5, MAD2L1, and ASPM, suggesting their pivotal role in the regulation of tumor development by SKA1/2/3 genes (Figure 6(d)).

### GO and KEGG analyses

**Figure 7.**
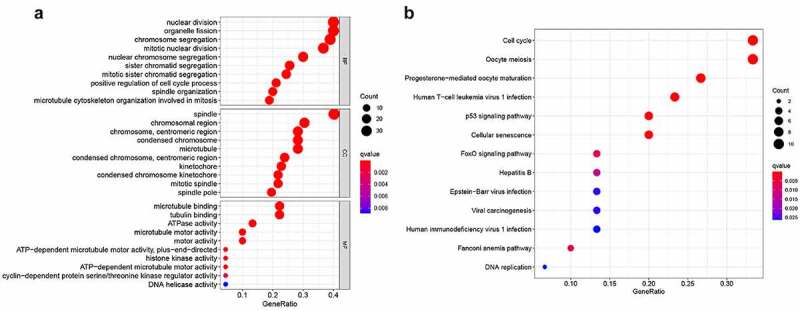
**GO functional and KEGG pathway enrichment analysis of genes co-expressed with SKA1/2/3**. (a) GO functional analysis revealing that co-expressed genes were most enriched in chromosome segregation, spindle and microtubule binding associated annotations. (b) KEGG pathway analysis revealing that co-expressed genes were most enriched in cell cycle, p53 signaling pathway, etc

To further identify the potential functions and signaling pathways of SKA1/2/3, we performed GO and KEGG analysis on co-expressed genes with SKA1/2/3. GO analysis was grouped into three parts: biological process, cellular component, and molecular function (Figure 7(a)). Biological process indicated that these genes were mainly involved in nuclear division, organelle fission, and chromosome segregation. Cellular component was commonly involved in spindle, chromosomal region, and chromosomal, centromeric region. Molecular function included microtubule binding, tubulin binding, and ATPase activity. KEGG analysis showed that SKA1/2/3 co-expression genes were primarily enriched in signaling pathways related to regulating cell cycle, oocyte meiosis, progesterone-mediated oocyte maturation, and p53 signaling pathway (Figure 7(b)). In summary, the above findings showed the latent mechanism of SKA1/2/3 in the carcinogenesis of BC, which established the direction for further research.

### Analysis of the expression and prognostic value of Hub genes in BC

**Figure 8.**
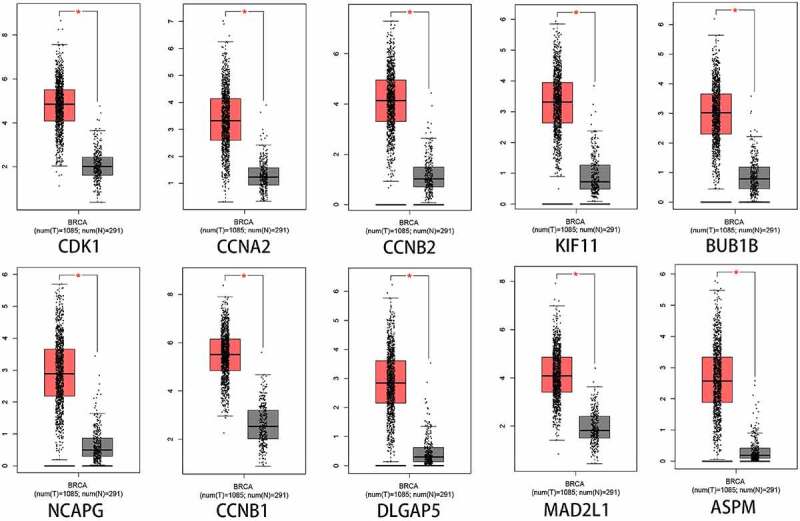
**10 Hub genes expression between normal and tumor tissues**. The expression levels of CDK1, CCNA2, CCNB2, KIF11, BUB1B, NCAPG, CCNB1, DLGAP5, MAD2L1, and ASPM in BC tissues were significantly higher than that in normal tissues

**Figure 9.**
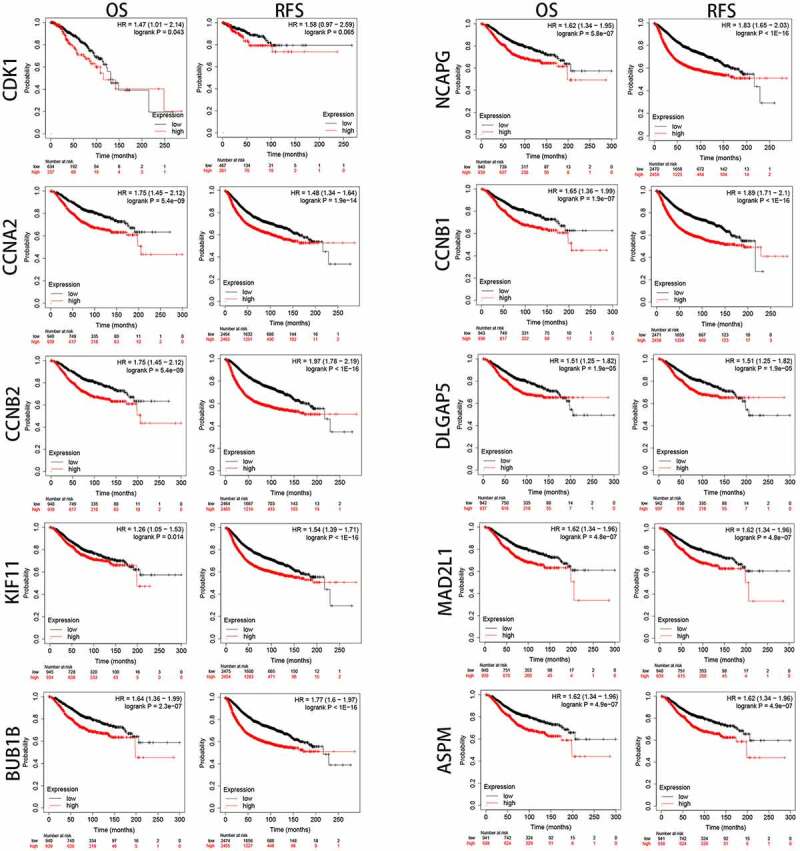
**The prognostic value of 10 Hub genes in BC patients**. CCNA2, CCNB2, KIF11, BUB1B, NCAPG, CCNB1, DLGAP5, MAD2L1, and ASPM were significantly associated with poor OS and RFS for patients with BC, whereas CDK1 was only associated with poor OS

To better evaluate the role of SKA1/2/3 in BC from multiple perspectives, we analyze the expression and prognostic value of 10 representative genes from the PPI network in GEPIA. As shown in Figure 8, the expression levels of CDK1, CCNA2, CCNB2, KIF11, BUB1B, NCAPG, CCNB1, DLGAP5, MAD2L1, and ASPM in tumor tissues were significantly elevated than in the normal tissues (P < 0.01). Moreover, CCNA2, CCNB2, KIF11, BUB1B, NCAPG, CCNB1, DLGAP5, MAD2L1, and ASPM were notably associated with poor OS and RFS of patients with BC, whereas CDK1 was only related to unfavorable OS (Figure 9) (P < 0.05).

### High SKA1/2/3 expression levels suggest an immunosuppressive microenvironment

**Figure 10.**
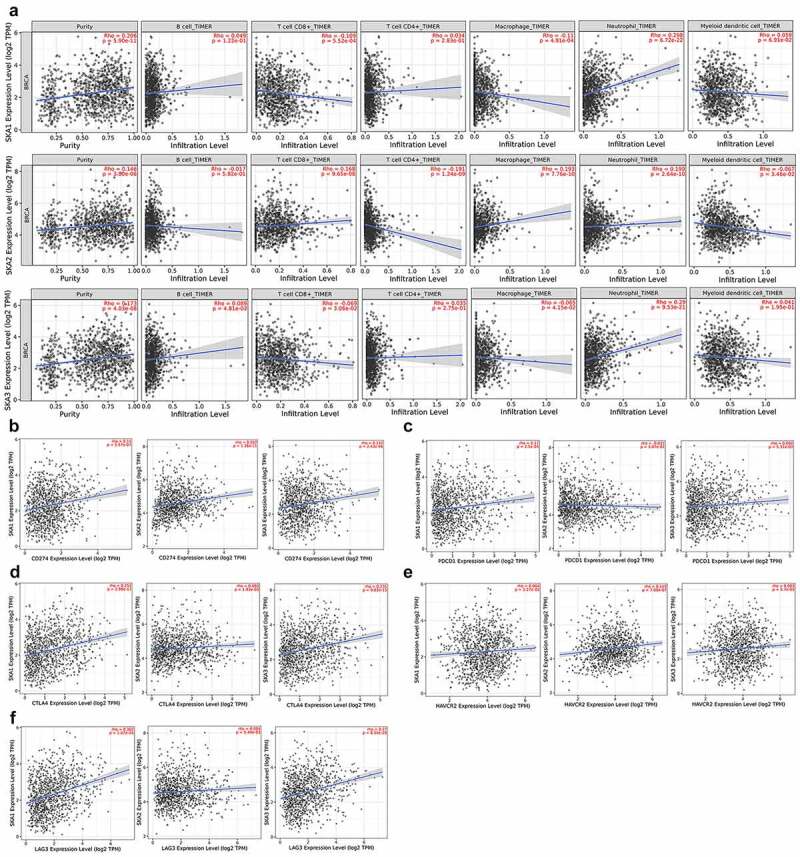
**High SKA1/2/3 expression levels suggest an immunosuppressive microenvironment**. (a) Correlations between the infiltration of immune cells and the expression of SKA1/2/3 in TIMER2.0. (b-f) Correlations between the expression levels of immune checkpoints (PDL1, PDCD1, CTLA4, TIM3 and LAG3) and SKA1/2/3

To further explore the immune cell infiltration in BC, scatter plot was conducted to visualize the association between B cells, CD8 + T cells, CD4 + T cells, Neutrophils, Macrophages, Dendritic cells infiltration, and tumor purity and SKA1/2/3 expression in the TIMER2.0 database (Figure 10(a)). Results reveal that the expression of SKA1 displayed a significant positive association with neutrophils infiltration and tumor purity, while a negative association with CD8 + T cells and macrophages infiltration (P < 0.05). Additionally, SKA2 expression exhibited a significantly positive association with the CD8 + T cells, macrophages, neutrophils infiltration, and tumor purity, and a negative association with CD4 + T and Dendritic cells infiltration (P < 0.05). Moreover, SKA3 expression was positively associated with the infiltration levels of B cell, neutrophils, and tumor purity, while negatively associated with the infiltration of CD8 + T cells and macrophages (P < 0.05).

Accumulating evidence suggests that the immune checkpoint plays an important role in the progression of tumor immune escape. As shown in Figure 10(b), the critical immune checkpoint (PD-L1, PDCD1, CTLA-4, TIM3, and LAG3), which protect the tumor from immune attack, were positively correlated with the expression levels of SKA1/2/3, except for PDCD1 which had no statistical difference with SKA2.

Thus, it can be seen that high expression of SKA1/2/3, to some degree, could contribute to the formation of an immunosuppressive microenvironment for patients with BC.

### Knockdown of SKA1/2/3 impeded the proliferation and migration of breast cancer

**Figure 11.**
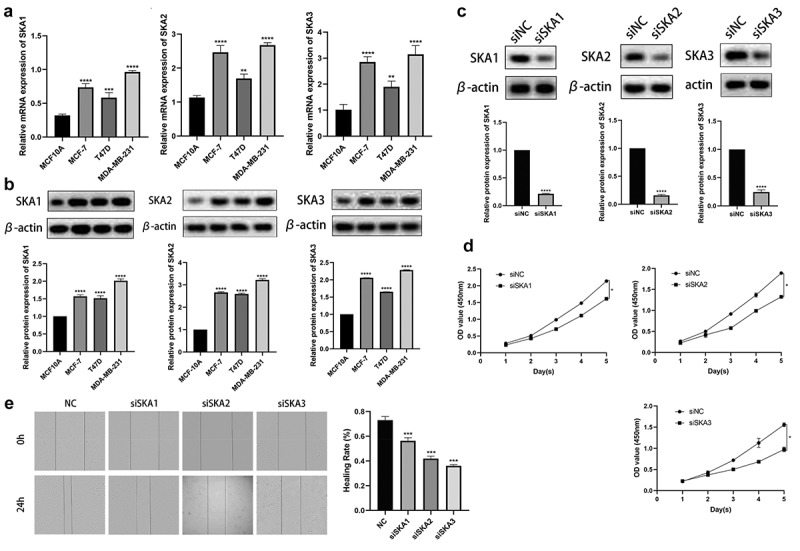
**SKA1/2/3 overexpression facilitates the proliferation and invasion of breast cancer**. (a) The mRNA levels of SKA1/2/3 were determined by RT-qPCR in normal (MCF10A) and BC cell line (MDA-MB-231). (b) Protein expression of SKA1/2/3 was monitored by western blot analysis in normal and BC cell line. (c) Western blot analysis was performed to verify the transfection efficiency of SKA1/2/3. (d) CCK-8 assays were used to evaluate the effect of SKA1/2/3 knockdown on cell proliferation. (e) The wound healing assay measured the effect of SKA1/2/3 on BC cell migration. *P < 0.05, **P < 0.01, ***P < 0.001, and ****P < 0.0001

To explore the molecular function of SKA1/2/3, we investigated the regulatory effect of SKA1/2/3 on the proliferation and migration of BC. First, we detected the expression levels of SKA1/2/3 mRNA and protein in a panel of BC cell lines by qRT-PCR and western blot, respectively. Consistent with the previous data, the experimental results showed that the expression of SKA1/2/3 at the mRNA and protein levels was markedly upregulated in the BC cell line compared with the normal control cell line (Figure 11(a,b)) and then the MDA-MB-231 cell was chosen for subsequent experiments. SKA1/2/3 siRNA were applied to deplete SKA1/2/3 expression among BC cells. The results of the western blotting analyses demonstrated that we have successfully knockdown SKA1/2/3 (Figure 11(c)). Notably, the CCK-8 assay demonstrated that knockdown of SKA1/2/3 caused a reduction in cell proliferation capacity (Figure 11(d)). In addition, the knockdown of SKA3 markedly suppressed the migration potential of BC cells (Figure 11(e)). Taken together, these data confirm that SKA1/2/3 play a promoting role in BC cell proliferation and migration.

## Discussion

It is well known that SKA family genes are involved in maintaining the stability of the mitotic process [17]. However, it has been widely reported in recent years that SKA family genes present abnormal expression and activation in multiple malignancies, such as cervical cancer, hepatocellular carcinoma, pancreas ductal adenocarcinoma, and gliomas, etc [6,18–20]. Although the role of SKA family genes in the occurrence and prognosis of many tumors has been recognized, its potential prognostic value and related biological functions in BC have not been fully understood. The present research aims to investigate the expression, mutation, prognosis, and immune correlation of SKA family genes in BC, and further analyze the co-expressed genes closely related to SKA family members in BC on the basis of the above studies to support our findings. Moreover, the results of the in vitro experiments further confirmed the oncogenic role of SKA family members in breast cancer. We expect to be able to increase the existing cognition of SKA family genes in the progression of BC through our study, thereby better serving future therapeutic strategies for tumors.

SKA1, a novel discovered gene, is the most studied gene in multiple cancer types among SKA family members. SKA1 was identified as the target of antitumor mir-10a-5p in renal cell carcinoma (RCC) and overexpression of SKA1 was associated with poor prognosis of patients with RCC. Moreover, the knockdown of SKA1 inhibited the migration and invasion of tumor cells [21]. As a tumor promoter of pancreatic ductal adenocarcinoma, SKA1 overexpression promoted tumor proliferation and migration in vivo and vitro through inhibiting G2/M arrest and regulating actin cytoskeleton organization by activating Cdc42^6^. It was also reported that high level of SKA1 was correlated with the progression and malignancy of non-small cell lung carcinoma (NSCLC), which also protects NSCLC cells from cisplatin-induced apoptosis [22]. In vitro experimental results suggested that SKA1 was overexpressed and knockdown of SKA1 inhibited proliferation and migration of BC cells. Moreover, SKA1 overexpression was correlated with poor OS, RFS, and DMFS. Interestingly, the results of database analysis demonstrate the positive correlation between SKA1 expression and tumor stage in HER2-positive BC and its guiding value for prognosis. Therefore, we speculate that SKA1 may be involved in the adverse development of breast cancer, especially HER2-positive BC, which is worthy of further study.

SKA2 functions as a tumor oncogene, participating in a multiplicity of important cellular mechanisms consisting of cell growth, metastasis, and cell cycle regulation. A previous report revealed that high level of SKA2 accelerated the proliferation, colony-forming, and invasion of hepatocellular carcinoma cells by means of upregulating Wnt/β-catenin signaling [7]. SKA2 overexpression reversed the miR-520a-3p-mediated inhibitory effect on the proliferative and invasive capacities of gastric cancer cells [23]. In addition, Dou et al [24]. showed that SKA2 and circ_0008039 were upregulated in cells and tissue of BC, while miR-140-3p was down-regulated. Moreover, SKA2 was confirmed to be a direct target of miR-140-3p, and silencing circ_ 0008039 positively regulated SKA2 via increasing miR-140-3p, thereby restraining the proliferation, invasion, and glycolysis of BC cells. The present study indicated that SKA2, which significantly correlated with the tumor stage, is highly expressed in BC. Subsequent experimentation revealed that SKA2, a candidate tumor-promoter, probably promotes BC cell proliferation and migration via upregulating SKA2 expression. However, high expression of SKA2 was only associated with RFS and MDFS, but not with OS and PPS. One possible reason for that may be intertumor heterogeneity, which needs more research to investigate.

SKA3 is a member of the SKA complex, which was considered to participate in malignant transformation in cancers. The study of Hu et al [20]. pointed out that upregulation of SKA3 elevated the expression of CDK4, p-Akt, Cdk2 cyclinE2, p-Rb, E2F1, and cyclinD1 in HeLa cells, thus promoting cervical cancer proliferation and migration. Besides, SKA3 overexpression facilitated proliferation and chemoresistance of laryngeal squamous cell carcinoma (LSCC) by reprogramming PLK1–AKT axis-mediated glycolytic metabolism, which was also related to the unfavorable prognosis of LSCC patients [25]. Similarly, the expression of SKA3 was upregulated in hepatocellular carcinoma tissues compared to adjacent tissues, and the knockdown of SKA3 can significantly accelerate cell apoptosis and inhibit tumor proliferation and invasion in vivo and in vitro [18]. Consistent with these studies, we showed that SKA3 presented high expression in BC and SKA3 expression was positively correlated with the clinicopathological feature. Besides, SKA3 overexpression was associated with patient prognosis including OS, RFS, PPS, and DMFS. Moreover, SKA3 silencing leads to a significant reduction in the proliferation and migration capacity of BC cells, indicating a central role of SKA3 as an oncogene in BC.

To prove the possible mechanism of SKA family genes in BC, we observed that the 10 Hub genes that are most closely related to SKA1/2/3 have a high guiding value for the occurrence and progression of BC. Go and KEGG analysis indicated that these genes were markedly enriched in regulating the cell cycle, p53 signaling pathway, and DNA replication. The cell cycle is a conservative evolutionary process that is crucial for cell growth. Dysregulation of the cell cycle is considered an important marker of tumors [26]. Notably, Cyclin-dependent kinase 1 (CDK1) was identified as an important protein regulating the cell cycle [27]. Previous studies demonstrated that activation and upregulation of CDK1 expression can promote triple-negative BC tumorigenesis [28]. Combined with studies that SKA3 mutant lacking CDK1 phosphorylation have deficits in kinetochore localization [29], we speculate that there may be a link between CDK1 and SKA3 in the regulation of BC progression, which requires further investigation to address this hypothesis. Moreover, Zhao et al. reported that knockdown of SKA1 reduced the expression of CCNB1 protein in adenoid cystic carcinoma [30]. However, the relationship between the remaining Hub genes and SKA1/2/3 was not discovered, and further tracking research is required. Some reports have elucidated that most of the Hub genes contribute to the progression of BC. Similar to our current results that all 10 Hub genes are not only highly expressed in BC, but also associated with poor prognosis.

Recently, immunotherapy has been generated as a novel pillar for cancer treatment. CD8 + T cells are considered to be a powerful immune prognostic marker for BC patients, especially in TN and HER2 + subtypes, and associated with a better prognosis in BC [31]. However, CD8+ and CD4 + T cells get a lower frequency in the tumor microenvironment, and their phenotype is related to immune failure [32]. Our study also showed that higher levels of SKA1/3 were associated with lower infiltration of CD8 + T cells, and so was the relationship between SKA2 and CD4 + T cells. Studies have shown that neutrophils increase the metastatic ability of tumors and deteriorate survival by inhibiting CD8 + T cells [33], NK cells [34], increasing the shedding of BC cells [35], and activating angiogenesis [33]. In our study, SKA1/2/3 expression was positively correlated with neutrophil infiltration. Macrophages represent key players in anti-tumor immunity through phagocytosis and antigen presentation, which also actively participate in the production and regulation of immune effector cells, as well as secretion of cytokines (e.g., TNF-α, IL-1, IL-6, and interferon (IFN)-α/β) [36]. Similar to macrophages, dendritic cells (DCs) play an integral role in the adaptive immune system owing to mature DCs cross-present antigen to CD8+ and CD4 + T cells, which stimulate them to eliminate tumor cells [37]. In this study, the expressions of SKA1/3 were negatively correlated with macrophages and SKA2 was negatively correlated with DCs.

Accumulating evidence suggests that immune checkpoint molecules on T-cells and tumor cells contribute to immune escape of tumor by facilitating tumor immunosuppressive effects [38]. Our data illustrated that among SKA1/2/3, except that SKA2 had no significant correlation with PDCD1, the rest were all positively related to PDL1, PDCD1, CTLA4, TIM3, and LAG3. Thus, these results implied that SKA1/2/3 may participate in the formation of an immunosuppressive microenvironment by decreasing cells with immunomodulatory effects, recruiting immunosuppressive cells, and synergistically up-regulating immune checkpoint molecules. Therefore, the implementation of combined immunotherapy appeared to be a promising choice for BC treatment.

In this study, we focused on analyzing the expression level of SKA1/2/3 in BC as well as the prognostic value for patients. Additionally, various correlations between SKA genes expression and different immune molecules, as well as levels of immune-cell infiltration of the tumor microenvironment, enhancing our understanding of the regulatory role of SKA genes on the tumor microenvironment. In vitro experiments on the functional role of SKA1/2/3 support their potential carcinogenic value in BC tumor. This study provides important insights that offer a direction for further in-depth exploration of the regulatory role of individual SKA genes in BC.

## Conclusion

Collectively, our current study revealed that SKA1/2/3 were upregulated and SKA1/3 overexpression was significantly correlated with unfavorable clinical outcome in BC patients. Furthermore, we comprehensively explored the potential possibility of SKA1/2/3 exerting a tumor immunosuppressive effect in BC patients. Furthermore, in vitro experimental verified the expression levels of SKA1/2/3 and its role in the proliferation and migration of BC cells. In general, SKA1/2/3 can serve as potential biomarkers and therapeutic targets for BC and may provide insight into the diagnosis and treatment of disease in the future.

## Data Availability

The datasets and materials of this study are derived from public databases, as follows. Oncomine database (www.oncomine.org), UALCAN database (https://ualcan.path.uab.edu/index.html) and Gene Expression Profiling Interactive Analysis (GEPIA) database (http://gepia.cancer-pku.cn/index.html), Kaplan-Meier Plotter database (https://kmplot.com/analysis/), cBioPortal database (www.cbioportal.org), The Cancer Genome Atlas (TCGA) (http://cancergenome.nih.gov/), String database (https://string-db.org/), TIMER2.0 database (http://timer.cistrome.org/).

## Abbreviations

BC: Breast cancer; SKA: spindle and kinetochore associated; OS: overall survival; RFS: relapse-free survival; PPS: post-progression survival; DMFS: distant metastasis-free survival; PFS: progress-free survival; DSS: disease-specific survival; TCGA: The Cancer Genome Atlas; GO: gene ontology; KEGG: Kyoto Encyclopedia of Genes and Genome; PPI: protein-protein interaction; DCs: dendritic cells; CDK1: Cyclin-dependent kinase 1; CCK-8: Cell Counting Kit-8; qRT-PCR: Quantitative real-time polymerase chain reaction
